# Alterations in serum microRNA in humans with alcohol use disorders impact cell proliferation and cell death pathways and predict structural and functional changes in brain

**DOI:** 10.1186/s12868-015-0195-x

**Published:** 2015-09-05

**Authors:** Cherry Ignacio, Steven D. Hicks, Patrick Burke, Lambert Lewis, Zsuzsa Szombathyne-Meszaros, Frank A. Middleton

**Affiliations:** Department of Biochemistry and Molecular Biology, SUNY Upstate Medical University, Syracuse, NY USA; Department of Pediatrics, SUNY Upstate Medical University, Syracuse, NY USA; Department of Psychiatry and Behavioral Sciences, SUNY Upstate Medical University, Syracuse, NY USA; Department of Neuroscience and Physiology, SUNY Upstate Medical University, 750 East Adams Street, Syracuse, NY 13210 USA; Developmental Exposure Alcohol Research Center, Binghamton University, Binghamton, NY USA

**Keywords:** Alcohol use disorders, microRNA, Biomarker, Next generation sequencing

## Abstract

**Background:**

There is currently a lack of reliable, minimally invasive biomarkers that could predict the extent of alcoholism-induced CNS damage. Developing such biomarkers may prove useful in reducing the prevalence of alcohol use disorders (AUDs). Extracellular microRNAs (miRNAs) can be informative molecular indicators of changes in neuronal gene expression. In this study, we performed a global analysis of extracellular miRNAs to identify robust biomarkers of early CNS damage in humans diagnosed with DSM-IV AUDs. We recruited a relatively young set of 20 AUD subjects and 10 age-matched controls. They were subjected to comprehensive medical, neuropsychological and neuroimaging tests, followed by comparison of miRNA levels found in peripheral blood serum. Employing a conservative strategy to identify candidate biomarkers, miRNAs were quantified using two independent high-throughput methods: microarray and next-generation RNA-sequencing. This improved our capacity to discover and validate relevant miRNAs.

**Results:**

Our results identified several miRNAs with significant and reproducible expression changes in AUD subjects versus controls. Moreover, several significant associations between candidate miRNA biomarkers and various medical, neuropsychological and neuroimaging parameters were identified using Pearson correlation and unbiased hierarchical clustering analyses. Some of the top candidate biomarkers identified, such as *mir*-*92b* and *mir*-*96* have established roles in neural development. Cross-species validation of miRNA expression was performed using two different in vivo rat drinking models and two different in vitro mouse neural stem cell exposure models. A systems level analysis revealed a remarkable degree of convergence in the top changes seen in all of these data sets, specifically identifying cell death, cell proliferation and cell cycle processes as most consistently affected. Though not necessarily the same molecules, the affected miRNAs within these pathways clearly influence common genes, such as p53 and TNF, which stand out as potential keystone molecules. Lastly, we also examined the potential tissue origins of these biomarkers by quantifying their levels in 15 different tissue types and show that several are highly-enriched in the brain.

**Conclusions:**

Collectively, our results suggest that serum miRNA expression changes can directly relate to alterations in CNS structure and function, and may do so through effects on highly specific cellular pathways.

**Electronic supplementary material:**

The online version of this article (doi:10.1186/s12868-015-0195-x) contains supplementary material, which is available to authorized users.

## Background

Alcohol use disorders (AUDs) are a major public health problem and impose a substantial socioeconomic burden. In the US alone, there is a one in seven lifetime prevalence of AUD [1]. Structural brain damage and the widespread alteration of neuronal function that accompanies it represent two of the most adverse effects of AUDs (recently reviewed in [2]). However, despite well-documented effects on the CNS, there are no definitive molecular signatures of AUD-induced brain damage. This limits the selection of rational interventions and hampers the ability to gauge therapeutic effects. Thus, developing biomarkers that indicate early CNS damage may prove useful in deterring the emergence of AUDs.

We recently completed an extensive messenger RNA (mRNA) expression-based study of human subjects with AUDs as well as adolescent and adult rats engaged in excessive alcohol consumption and mouse neural stem cells (NSCs) exposed to ethanol in vitro [3]. In that study, we sought to identify mRNA biomarkers in peripheral blood leukocytes (PBLs) that could predict CNS dysfunction related to excessive alcohol consumption across different species and models. Based on pre-existing data, we chose to test only a specific set of mRNAs with functions related to cell cycle regulation, DNA damage and repair, as well as p53 signaling. We found that a subset of the tested mRNAs was consistently changed in human AUD subjects, ethanol-consuming rats, and ethanol-exposed NSCs. Moreover, several of the affected mRNAs showed robust correlations with different biological variables, including neuroimaging volumes, neuropsychological performance scores, as well as indices of ethanol consumption.

Despite promising leads from our focused mRNA study, there were a number of limitations on its overall potential utility. First, because we examined expression of mRNA from the PBLs of our AUD subjects, we could only speculate about potential transcript changes in other tissues, such as the brain. Second, many subjects in that study had long histories of AUD, and therefore did not represent an optimal subject set for discovering markers of early AUD-induced alterations, which could potentially be reversible. Third, the analysis we performed was focused exclusively on a limited set of a few hundred mRNAs that had been identified through previous screens [4] rather than the entire transcriptome. Finally, the alterations in brain volumes we observed in the previous study were not controlled for overall brain shrinkage, which is well-characterized in AUDs as well as advanced aging.

Because of the limitations just described, in the present study, we sought to expand upon our previous work by examining whether we could identify even more robust biomarkers using: (1) a younger subset of AUD subjects with shorter histories of alcohol consumption who were completely age-matched to controls; (2) neuroimaging measures that were normalized to whole-brain volumes; and (3) measurements of extracellular microRNAs (miRNAs) rather than leukocyte mRNA for detection of early stage CNS damage.

Our current focus on miRNAs builds upon considerable recent interest in their potential use as biomarkers in other diseases and conditions. MicroRNAs are short, hairpin-derived RNAs that repress protein expression of a large fraction of the genome in a vast array of species. In mammals, approximately half of all mRNAs maintain selective pairing with miRNAs [5]. Thus, miRNAs have emerged as a class of master regulatory molecules that control the level of post-transcriptional gene expression. In neurons, miRNAs not only regulate mRNA levels, but they also compartmentalize specific mRNA expression within subcellular regions such as axons and dendrites [6].

Dysregulation of brain miRNAs have also been recently associated with alcohol exposure. In human post-mortem brain samples, robust changes in miRNA expression have been reported in the prefrontal cortex of subjects with a history of chronic alcohol abuse compared to controls [7]. Robust changes in miRNA levels have also been reported in the brains of rats following alcohol intoxication [8]. These findings are further supported by in vitro studies of the effects of ethanol effects on miRNA levels in neurosphere cultures [9].

In addition to their intracellular roles, miRNAs are released by normal and damaged cells into the bloodstream and can serve as conduits for the spread of genetic and pathological information to distant cells and tissues. In this manner, miRNAs have been shown to mediate the spread of cell and tissue damage or alter the microenvironment [10]. In fact, brain-specific miRNAs have been identified in the blood and other bodily fluids and it has been suggested that they may provide the opportunity to evaluate ongoing changes in the CNS upon the initiation of neurodegeneration [11]. For example, in Alzheimer’s disease, miRNAs that are associated with neuropathological changes in post-mortem brain tissue have been detected in blood sera of subjects meeting ante-mortem criteria for the disease, albeit at lower basal levels [12]. The potential importance of such findings gains further support from a recent study that demonstrated serum mRNA levels could be directly regulated by brain-specific administration of RNAi [13].

In this study, we compared miRNA expression levels in the serum of human subjects diagnosed with AUDs to those in healthy control subjects in order to identify potential biomarkers of CNS alterations. Further, we explored the potential relationship of candidate biomarkers with various medical, neuropsychological and normalized neuroimaging data. Cross-species validation through miRNA profiling of various in vivo and in vitro ethanol exposure paradigms helped confirm key miRNA molecules affected by ethanol and their mRNA targets. Taken together, our data provide striking new insight into the potential epigenetic regulation of CNS gene expression alterations brought about by AUDs.

## Results and discussion

In this study, we determined the feasibility of using serum miRNAs to predict the extent of structural and functional CNS impairments associated with alcohol use disorders. The subjects recruited for this study were selected to reflect the same average age and an equal number of males and females in each group (Table 1). Compared to our previous study that explored the use of mRNA profiles from peripheral blood leukocytes [3], the current study focused on a younger group of individuals, allowing exploration of potentially more sensitive biomarkers of early CNS alterations. Such early detection could promote development of therapeutic and preventative interventions that might halt or potentially reverse the CNS effects.

**Table 1:** Human subject characteristics

	Control (n = 10)	AUD (n = 20)	DSM-IV diagnosis	
			AA (n = 4)	AD (n = 16)
Gender	5F, 5M	10F, 10M	2F, 2M	8F, 8M
Age (years)	30.9	30.9	26.8	31.9
Drinking days last month	–	15.3	15.25	15.31
Drinks/drinking day	–	5.1	4	5.4
Heavy drinking days last week	–	1.2	1	1.25
Age at onset	–	18.6	19.5	18.3
Years drinking	–	12.4	7.25	13.6

AUD and control subject demographics. Both groups reflect the same average age. Subjects with alcohol use disorders were diagnosed using the DSM-IV alcohol abuse and dependence diagnostic criteria. A heavy drinking day was defined as 4 standard drinks for women and 5 for men

On average our AUD subjects had been drinking an average of 12 years, starting around age 18. They drank alcohol an average of 15 days during the month prior to recruitment, and consumed approximately five drinks per drinking day. As a group, the AUD subjects had an average of one heavy drinking day (i.e. more than five drinks for men or four drinks for women) during the week prior to recruitment.

### AUD subjects show changes in specific brain regions, and a subset of clinical, medical and neuropsychological measures

Comprehensive demographic, medical, neuropsychological and neuroimaging data were collected from each of the subjects. Prior to hypothesis testing, variables were examined for equality of variance in AUD subjects and controls using a Fisher’s F test. Variables that passed the F test (p-value > 0.05) were examined for differences between AUD subjects and controls using a Student’s T-test, while those with unequal variance were tested using a Welch’s T-test. Unless otherwise stated, we did not correct for multiple testing due to the relatively small sample size and the desire to identify as many potential biomarkers as possible in our initial parametric screen. These were subsequently examined for quantitative associations with the other variables of interest.

#### AUD subjects show elevations in GGT

Our statistical screen identified a relatively small set of variables that differed between AUD subjects and controls (Table 2). Among the standard medical laboratory blood tests (including several that assess liver function), only the gamma-glutamyl transferase (GGT) assay differed between AUD and control subjects, with an average increase of 85 %. Notably, the average level in AUD subjects was 37.4 IU/L, which is above the normal range for males and females of the same age as our subjects. These elevated levels suggest at least some amount of early liver damage may be occurring in our subjects, since GGT elevations are commonly seen in patients with alcoholic liver disease, a well-documented effect of chronic alcohol consumption.

**Table 2:** Medical, neuropsychological, and neuroimaging variables with significant changes in AUD subjects

Variable	% Δ AUD	T-test p-value	Equality of variance
Medical			
Gamma-glutamyl transferase^a^	84.9	0.040	0.001
Neuropsychological			
Letter fluency score^a^	−21.8	0.017	0.035
Normalized neuroimaging volumes			
Cerebrospinal fluid	31.7	0.005	–
Anterior corpus callosum	−14.6	0.008	–
Left temporal superiorlateral gyrus	−10.4	0.010	–
Left ventral diencephalon	6.9	0.011	–
Left central sulcus	16.7	0.012	–
Left cingulomarginal sulcus	−11.9	0.015	–
Right temporal superiorlateral gyrus	−10.7	0.019	–
Right pars orbitalis white matter	13.3	0.024	–
Left inferior occipital gyrus and sulcus	−14.9	0.026	–
Left superior temporal cortex	−7.0	0.027	–
Right anterior collateral transverse sulcus	−15.4	0.032	–
Left Brodmann’s area 3a	12.7	0.032	–
Left superior frontal cortex	8.0	0.038	–
3rd ventricle	22.3	0.039	–
Right intraparietal and posterior transverse sulcus	−10.3	0.041	–
Right pars opercularis	−13.2	0.043	–
Right superior parietal cortex	−8.4	0.043	–
Left inferior frontal triangular gyrus	−16.0	0.044	–
Right inferior parietal cortex	−7.7	0.049	–
Right cingulomarginal sulcus	−8.4	0.050	–

Comparison of various clinical and imaging parameters between AUD and control subjects. Neuroimaging measurements were normalized to whole brain size to correct for overall brain shrinkage before calculating  % changes relative to values from controls

^a^Indicates variables determined to have unequal variances using the Fisher’s F test that were examined for differences between groups using the Welch’s T-test. All other variables were evaluated using the Student’s T-test

#### AUD subjects show reductions in verbal fluency

Despite the large number of neuropsychological tests administered, AUD subjects only showed significant differences compared to controls in the Letter Fluency Test of the Delis-Kaplan Executive Function System (D-KEFS) [14]. This test examines the ability to rapidly generate non-redundant words that begin with specific letters in a short time interval. The AUD subjects scored 22 % worse on average than controls in their scaled scores, indicating a relative impairment of a specific language function. This observation is consistent with significant verbal fluency abnormalities that have been reported in patients with much longer histories of alcohol use disorders, including those with Korsakoff syndrome, a severe neurologic complication of AUD [15].

The subtlety and specificity of our neuropsychological findings may reflect the younger average age of our subjects, where alcohol-related brain damage phenotypes are more difficult to detect. Comparisons of our results are most appropriately made to neuropsychological studies of adolescent and young adult drinkers. Letter fluency tests on university age (18–20 years) subjects have not found differences in performance when binge drinkers were compared to non-binge drinkers [16]. However, longer-term studies on adolescent alcohol abusers (13–19 years, followed up after 4 years) have displayed significantly impaired language skills [17]. This suggests that the language impairment we observed manifests itself later in young adulthood since our AUD subjects typically started drinking during adolescence (average onset of drinking age: 18.6 years). Indeed, our AUD subjects showed a significant correlation between Letter Fluency Test score and the number of years drinking (R = −0.46, p < 0.05). Although the number of years drinking was confounded with age in our AUD subjects, we did not observe a significant association between age and Letter Fluency Test score in our controls.

#### AUD subjects exhibit overall loss of gray matter and increased sulcal size

**Fig. 1:**
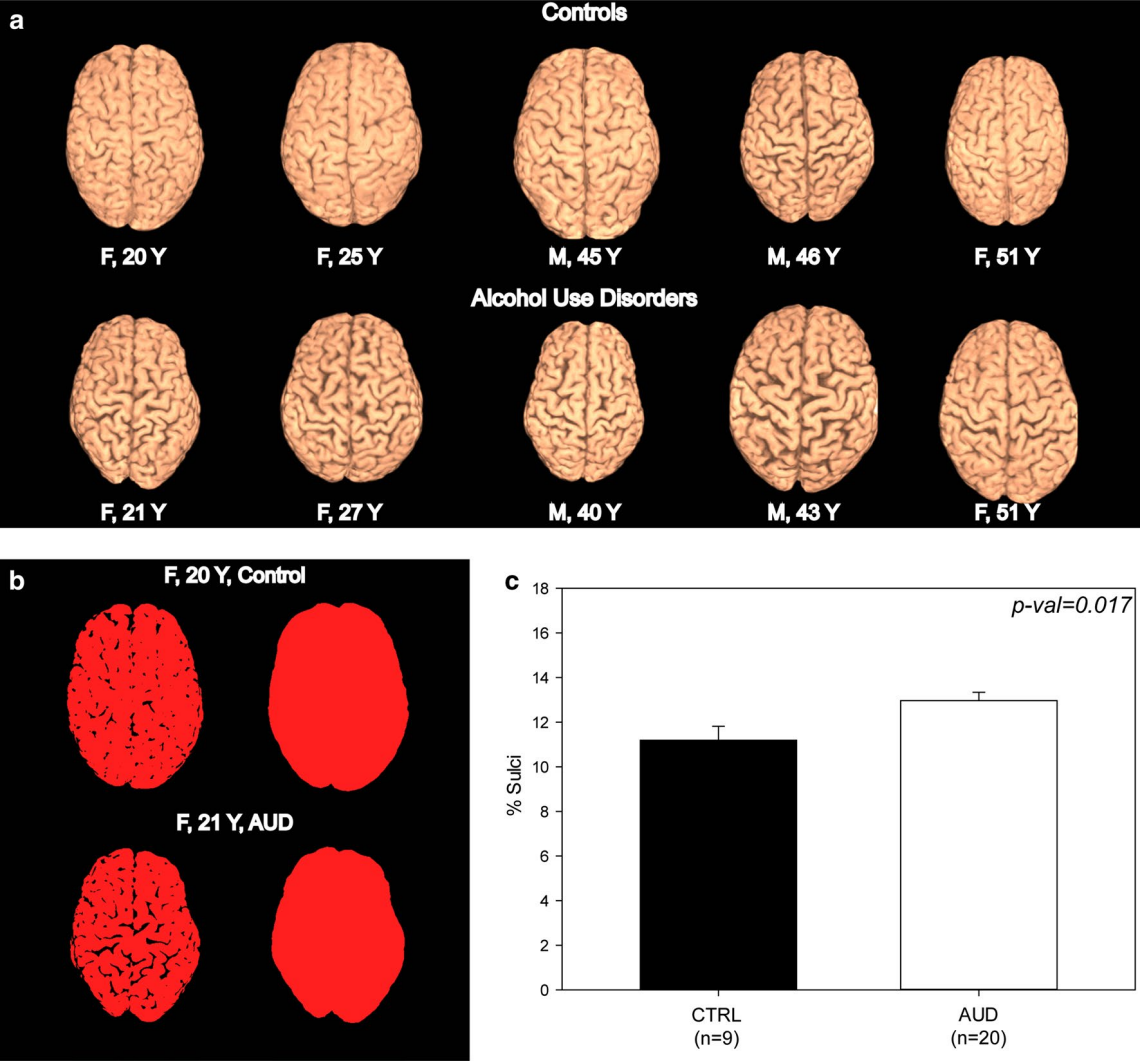
Subjects with alcohol use disorders have increased sulcal size. **a** Skull-stripped brain reconstructions show increased sulcal size in subjects with AUDs when compared to age- and gender-matched controls. **b** Sulcal size was measured in each reconstruction and expressed as a percentage of the brain’s area. **c** Plot of mean sulcal size in AUD and control subjects (±SEM). Group differences in % sulci between subjects with AUDs and controls were determined using a Student’s T-test. *Y* years of age

Screening for possible structural brain changes in AUD subjects versus controls was initially performed using a combination of two different autothresholding methods on skull-stripped 3D reconstructions of MRI data (Fig. 1a). The moments method [18] created a binary image which excluded the sulci while the triangle method [19] created a binary image that included sulci and thus encompassed the entire visible external brain surface. The difference between these two images indicated the percentage of visible surface area occupied by cortical sulci (Fig. 1b). A Student’s T-test performed on these measurements indicated significantly larger sulcal size (16 %, p = 0.017) in AUD subjects (Fig. 1c).

#### AUD subjects exhibit volumetric changes in multiple brain regions

Having observed an increased sulcal size even in this relatively young cohort of AUD subjects, we further examined possible differences in regional brain volumes using a comprehensive unbiased approach to measure more than 360 pre-defined brain regions with the FreeSurfer software suite [Reviewed in 20]. Moreover, in contrast to our previous volumetric analysis of older AUD subjects and controls [3], we normalized all of these measurements to total brain volume in order to control for overall brain shrinkage that occurs with AUD or aging. In this manner, we hoped to find changes indicative of enhanced vulnerability to AUD in some brain regions in a younger cohort. The subsequent comparisons between AUD and control subjects yielded 20 individual regions with volumes that differed between groups (Table 2). Notably, because of our normalization method, the magnitude of changes in these 20 regions can be considered greater than that of the entire brain.

Consistent with our initial sulcal measurements, the FreeSurfer-based volumetric analysis supported widening of the left central sulcus as well as ventricular enlargement in the 3rd and 4th ventricles and an increase in total CSF volume. Four out of the seven regions representing ventricular and CSF spaces as well as sulci were larger in our AUD subjects. These findings support previous research showing greater than normal ventricular enlargement and sulcal widening in relation to increasing age [21] as well as CSF size [22].

Most of the remaining regional volumes showed a decrease in AUD subjects, consistent with their enhanced shrinkage relative to overall brain volume reduction. Indeed, alcohol-related brain damage (ARBD) has been well documented and does demonstrate some regional specificity, although some of these changes may be reversible by abstinence [Reviewed in 23]. Examples of such regions include the anterior corpus callosum, which we observed to be almost 15 % smaller in our AUD subjects, consistent with a previous study of male [24] and female alcoholics [25]. Notably, however, the average ages of participants in the studies from that group (Males: 48.8 ± 10.7 years; Females: 40.9 ± 9.6 years), were a decade older than our subjects (30.9 ± 10.2 years), suggesting that corpus callosum volume shrinkage is occurring earlier than previously thought.

### Global, high-throughput human miRNA expression screening and verification

To ensure optimal sampling, we employed a conservative strategy to identify and verify potential miRNA biomarkers Quantification of miRNAs were based primarily on next-generation small RNA-sequencing (RNA-Seq), which has emerged as a highly accurate method of miRNA quantification owing to its sensitivity and considerable dynamic range [26].

To validate our RNA-Seq findings, and help reduce the type I (false positive) error rate, we used Affymetrix miRNA GeneChips as a complementary analysis. The combined application of these two technologies improved our capacity to discover AUD-relevant miRNAs that would have been overlooked had a single quantification method been employed. Indeed, we have recently used and discussed the merits of this combined approach in our studies of miRNA changes in adolescent rat brain following fetal alcohol exposures [27].

#### Most miRNAs in serum are present in fully mature -5p or -3p forms

Initial serum miRNA quantification was performed by aligning the Illumina RNA-Seq data to the hg19 human reference genome. This was performed because most miRNAs are encoded in intergenic regions, within non-coding RNA genes or the introns of protein-coding genes. These primary miRNA transcripts (pri-miRNAs) are transcribed and processed in the nucleus before being exported out of the nucleus in their precursor hairpin forms (pre-miRNA). Further processing in the cytoplasm via the protein Dicer creates mature single-stranded miRNAs of approximately 20 nucleotides. These mature miRNAs then assemble into an RNA-induced silencing complex (RISC) that interacts with complementary messenger RNAs, and either target them for degradation or interfere with translation efficiency [Reviewed in 28].

**Fig. 2:**
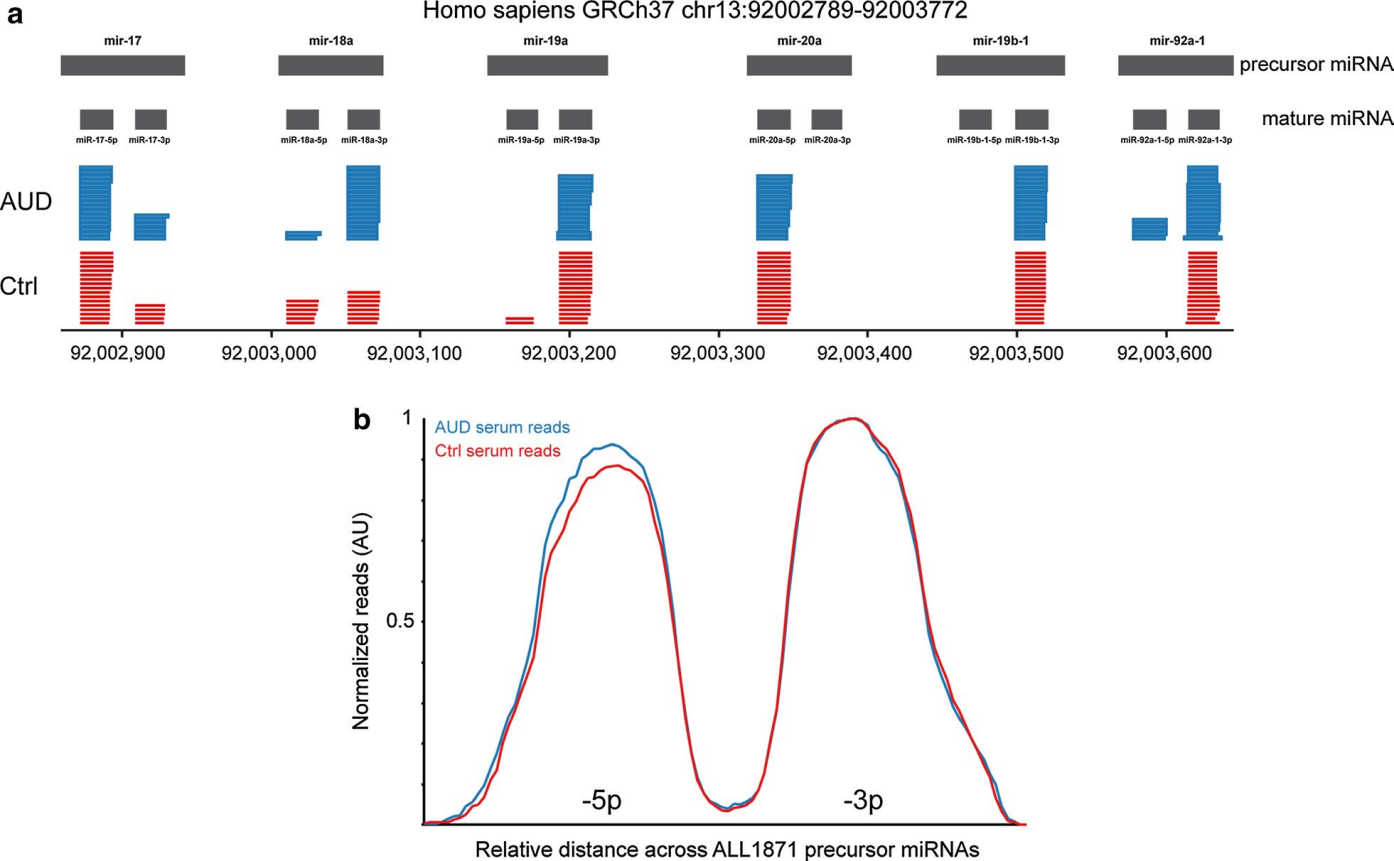
MicroRNAs in serum are in mature form. **a** Representative image showing RNA-seq reads aligned to region on chromosome 13. Reads are concentrated on mature miRNA chromosome coordinates as defined by the miRBase 20 database. **b** Trend plots mapping reads across all precursor miRNAs in the human genome show that reads are concentrated in regions that roughly correspond to mature miRNAs (-5p or -3p regions). We point out that the data shown are merely illustrative of the alignments produced during analysis of the RNA-Seq reads. Note that mature miRNAs are derived from the same strand as the full-length precursor, and appear in the location specified by the -5p box or -3p box. Also note that while the absolute levels of some mature miRNAs appeared to differ between AUD and Control subjects, the pattern of -5p or -3p usage appeared to be highly similar, which is best appreciated for the entire set of data in the *lower plot*

After whole genome alignment, we quantified reads using the RefSeq transcript annotation database and miRBase, an established, high-confidence database of microRNAs [29]. The latter quantified both precursor and mature miRNA regions. Inspection of our aligned reads against mature and precursor miRNA gene regions demonstrated that most reads were highly concentrated in one of two regions, corresponding to the mature -5p or -3p regions of individual miRNAs, with very few reads mapping to the flanking or internal sequence regions that would be derived from immature pre-miRNA or pri-miRNA alignments (Represented by Fig. 2a). Indeed, a probe trend plot of all 1871 primary miRNA transcripts available in miRBase 20 showed a striking bimodal distribution (Fig. 2b), and strongly suggested that the majority of miRNAs we quantified in serum were present in a mature (i.e., fully processed and possibly bioactive) form. Moreover, while the absolute levels of some miRNAs did appear to differ between subject groups, the overall pattern of preference for -5p or -3p mature forms was highly similar.

#### A tier system for classifying between-group differences across platforms

After alignment, we used the same statistical testing method described for the medical, demographic and neuroimaging variables to compare the miRNA levels between subject groups for both the RNA-Seq and microarray data sets. Individual miRNAs with were then assigned to two different classification tiers depending on the strength of evidence for concordant changes in gene expression across the two quantification platforms (Table 3). Because of the greater sensitivity and dynamic range of RNA-Seq, the values produced using this method formed the primary basis for comparison of the subject groups, with differences detected through the microarrays serving as a means of cross-platform validation. Tier A molecules represented stringent, nominally significant (p < 0.05) miRNAs generated from RNA-Seq quantification that also showed a strong trend (p < 0.10) and directionally consistent changes in the microarray experiments. Tier B molecules represented nominally significant (p < 0.05) miRNAs as determined by RNA-Seq that showed similar directional changes in the array data regardless of the p value. When possible, the mature forms of the miRNAs were listed within each Tier (these contain a capital R in miR- and a -3p or -5p in the title, according to established convention). Notably, for miRNAs in Tiers A and B, the overall correlation in the percent changes seen in AUD subjects with microarrays and RNA-Seq was 0.588 (p < 0.017).

**Table 3:** Most consistently changed miRNAs in AUD

Symbol	RNA-seq							Microarray	
	miRBase acc#	miRBase family	MiRBase fam acc #	Ctrl RPM	AUD RPM	% Δ AUD	P-value	% Δ AUD	P-value
Tier A: RNA-seq p < 0.05, Array p < 0.1, consistent % Δ AUD									
mir-96	MI0000098	mir-96	MIPF0000072	4.6	11.3	142.6	0.031	15.0	0.059
mir-320b-1	MI0003776	mir-320	MIPF0000163	61.0	144.6	137.1	0.004	22.2	0.071
mir-1976	MI0009986	mir-1976	MIPF0001633	4.1	9.9	138.1	0.039	24.1	0.081
Tier B: RNA-seq p < 0.05, consistent % Δ AUD									
mir-24-1	MI0000080	mir-24	MIPF0000041	31.3	90.5	189.4	0.008	6.2	0.338
mir-30a	MI0000088	mir-30	MIPF0000005	108.2	209.9	94.0	0.014	5.1	0.860
mir-92b	MI0003560	mir-25	MIPF0000013	7141.9	4553.8	−36.2	0.016	−8.2	0.312
miR-96-5p	MIMAT0000095	mir-96	MIPF0000072	111.7	252.0	125.5	0.018	8.0	0.455
mir-127	MI0000472	mir-127	MIPF0000080	68.6	158.9	131.5	0.021	8.4	0.455
mir-136	MI0000475	mir-136	MIPF0000099	4.4	9.7	121.2	0.035	1.5	0.760
miR-301a-3p	MIMAT0000688	mir-130	MIPF0000034	205.9	85.1	−58.7	0.045	−2.4	0.878
mir-320b-2	MI0003839	mir-320	MIPF0000163	61.3	148.8	142.9	0.004	5.2	0.333
mir-421	MI0003685	mir-95	MIPF0000098	15.5	30.3	95.6	0.020	9.0	0.725
miR-660-5p	MIMAT0003338	mir-188	MIPF0000113	196.9	99.7	−49.4	0.028	−10.2	0.451
mir-671	MI0003760	mir-671	MIPF0000358	14.8	44.4	200.0	0.003	4.2	0.692
mir-3615	MI0016005	mir-3615	MIPF0001540	50.1	78.3	56.4	0.031	1.3	0.881
mir-3676	MI0016077			2.1	11.1	437.1	0.027	12.9	0.218

RNA-sequencing of human serum samples determined miRNAs with nominally significant alterations and trends in patients with AUDs. Comparison with microarray data subsequently identified changes that were consistent across platforms. MicroRNAs were classified into two tiers based on concurrence of both analyses. Where available, microRNA families for each miRNA are also listed. Note that these are nominal (uncorrected) p values

*RPM* normalized reads per million

#### Most changed miRNAs are increased in AUD serum

Examination of the miRNAs that were most robustly changed in our AUD subjects indicated that most were increased in expression (Table 3). This observation is consistent with miRNA findings in post-mortem brains of human alcoholics [7]. Thus, the increased miRNA we detected in AUD subjects suggests a possible compensatory mechanism for neuronal injury, or potentially even enhanced transport of miRNAs from the brain to the periphery as a result of cell damage. Similar findings for other neurodegenerative conditions such as traumatic brain injury have also been reported [30]. A total of 3 miRNAs in Tier A and 13 in Tier B were identified (Table 3). Interestingly, several of these miRNAs have been previously found to be present at abnormal levels in studies of post-mortem human alcoholic brain tissue and animal models of ethanol exposure. For example, we found evidence of decreased *mir*-*92b* and increased *mir*-*96, mir*-*24, and mir*-*136* in our AUD subjects, consistent with studies of primary cortical neurons [31], mouse frontal cortex [32] and rat ventral striatum [27, 33]. Moreover, some of the miRNAs that we observed to change in the serum of AUD subjects were also shown to change in the same direction in studies of hepatocytes from mice with alcoholic liver damage (*mir*-*127*; see [34]) or whole zebrafish embryos exposed to alcohol (*mir*-*24*; see [35]).

On the other hand, we also observed some changes in the serum of AUD subjects that were opposite those reported in other studies. For example, we observed decreased expression of *mir*-*301* (Tier B; Table 3), while other studies have shown increased levels in various brain regions after alcohol exposure. This includes our own findings in the amygdala [27] of prenatally-exposed rats, as well as the prefrontal cortex [8, 32] of postnatally-exposed mice, and the frontal cortex from post-mortem brains of human alcoholics [7]. Such discrepancies could merely reflect the variability of different tissues being analyzed, but it is also possible that the levels reported in those studies are greatly influenced by intracellular miRNA processing, whereas the present data are derived from more stable extracellular serum miRNAs.

One miRNA finding of particular interest is *mir*-*30a*, which we observed to be increased 94 % in AUD subjects (Tier B; Table 3). Although *mir*-*30* was found to be decreased in whole embryos after ethanol exposure [35], it has been consistently shown to be increased in the brain of various models of ethanol exposure including whole brain [36], nucleus accumbens [33] and frontal cortex [32, 37]. Moreover, through its regulation of BDNF expression, *mir*-*30a* overexpression in the frontal cortex has also been shown to promote excessive alcohol intake which can then be reversed by inhibition of miRNA activity [37].

### Association of serum miRNA levels with neuropsychological, demographic and neuroimaging variables identifies a third tier of miRNAs

We integrated our clinical and miRNA expression datasets to identify potentially relevant associations which could be further explored as indicators of AUD. This was examined using a Pearson correlation analysis to compare miRNA expression levels and nominally significant neuropsychological, medical and neuroimaging measurements. These comparisons specifically did not include correlations between brain regions or between miRNA levels, which would be very strong, but not particularly informative for this purpose. Notably, indices of alcohol consumption were only included in the association testing of AUD subjects (since our control subjects did not drink) in an effort to identify direct biomarkers of alcohol consumption. Pearson correlations were used to calculate T-scores and corresponding P-values. Variables that were changed in our parametric analyses (Tables 2, 3) were not corrected for multiple testing when evaluating their correlations. The results of this association testing are shown in Table 4. We also performed an exploratory screen of all expressed miRNAs against all other variables in order to detect associations in the AUD subjects that may have eluded detection in the parametric two group comparison. These correlations were not guided by any predictions about possible directional differences and thus were corrected for multiple testing using the Benjamini-Hochberg False Discovery Rate (FDR) algorithm. Variables with an FDR <0.05 in the AUD subjects were defined as Tier C miRNAs and displayed along with the R, P, and FDR values observed in the Control subjects and combined subject groups (Table 5).

**Table 4:** Variables correlated with Tiers A or B miRNAs in AUD subjects (p < 0.05)

miRNA	Variable	AUD			Ctrl			All	
		R	P-value	N	R	P-value	N	R	P-value
In Tiers A and B									
miR-660-5p	Drinking days last month	0.626	0.030	6					
mir-421	Drinking days last week	0.482	0.020	12					
mir-320b-1	Drinks per drinking day	0.599	0.007	15					
mir-320b-2	Drinks per drinking day	0.934	0.002	15					
mir-24-1	Gamma-glutamyl transferase	0.448	0.034	16	−0.558	0.276	9	0.312	0.084
mir-92b	Gamma-glutamyl transferase	0.691	0.005	17	0.306	0.329	10	0.173	0.350
mir-421	Gamma-glutamyl transferase	0.476	0.022	14	−0.396	0.404	9	−0.024	0.914
mir-671	Gamma-glutamyl transferase	0.594	0.043	16	−0.016	0.968	9	0.538	0.001
mir-1976	Gamma-glutamyl transferase	0.474	0.023	9	0.595	0.091	7	0.509	0.017
mir-3615	Gamma-glutamyl transferase	0.476	0.028	17	−0.002	0.997	10	0.046	0.817
mir-24-1	Left inferior frontal triangular gyrus	0.548	0.006	16	0.547	0.068	9	0.060	0.768
mir-136	Left inferior frontal triangular gyrus	0.567	0.005	7	0.211	0.708	5	−0.108	0.753
mir-1976	Left inferior frontal triangular gyrus	0.531	0.009	9	−0.069	0.887	7	−0.327	0.307
mir-320b-2	Left ventral diencephalon	0.930	0.002	17	−0.606	0.213	10	−0.229	0.311
miR-301a-3p	Letter fluency score	0.732	0.025	6	0.797	0.055	4	0.480	0.077
mir-320b-2	Letter fluency score	0.771	0.032	17	−0.396	0.371	9	−0.467	0.065
miR-660-5p	Letter fluency score	0.602	0.040	6	0.700	0.114	4	0.753	0.001
mir-30a	Right anterior collateral transverse sulcus	0.467	0.026	17	0.080	0.819	10	−0.240	0.292
miR-301a-3p	Right anterior collateral transverse sulcus	0.845	0.005	6	0.652	0.152	5	0.728	0.002
mir-92b	Right cingulomarginal sulcus	0.547	0.037	17	0.209	0.524	10	0.390	0.019
mir-24-1	Right pars opercularis	0.441	0.037	16	−0.009	0.983	9	−0.099	0.655
mir-136	Right pars opercularis	0.447	0.034	7	−0.556	0.496	5	−0.287	0.443

Pearson correlation analysis of miRNA expression levels in AUD subjects reveal significant relationships between specific miRNA expression levels and other medical, drinking and neuroimaging variables that were significantly different in AUD subjects

*R* Pearson correlation coefficient, *N* number of pairs used in the calculation

**Table 5:** Tier C variables significantly correlated with any miRNA in AUD subjects (FDR < 0.05)

miRNA	Variable	AUD				Ctrl				All		
		R	P-value	BH FDR	N	R	P-value	BH FDR	N	R	P-value	BH FDR
In all miRNAs												
let-7a-5p	Heavy drinking days last week	0.846	3.1E−06	0.034	9							
mir-93	Heavy drinking days last week	0.869	1.3E−07	0.006	10							
miR-139-5p	Heavy drinking days last week	0.992	2.2E−06	0.048	4							
miR-1180-3p	Heavy drinking days last week	0.975	5.07E−06	0.043	3							
miR-584-5p	Left lateral fusiform gyrus	0.804	4.03E−06	0.040	17	−0.524	0.264	1	10	0.421	0.011	0.541
miR-16-2-3p	Left precuneus	0.977	4.38E−06	0.040	8	−0.045	0.925	0.995	7	0.540	0.013	0.577
mir-5010	Left subparietal sulcus	0.934	4.22E−07	0.014	12	0.729	0.026	0.988	7	0.807	7.80E−07	0.038
miR-4433-3p	Right insular white matter	0.948	2.59E−06	0.047	10	0.004	0.992	1	8	0.309	0.157	1
miR-4433b-5p	Right insular white matter	0.948	2.59E−06	0.047	10	0.004	0.992	1	8	0.309	0.157	1
mir-378a	Right medial orbital olfactory sulcus	0.821	1.85E−06	0.046	17	−0.268	0.520	1	10	0.463	0.004	0.597
miR-150-3p	Right paracentral white matter	0.830	2.78E−06	0.034	16	0.724	0.015	0.616	8	0.702	4.61E−06	0.062
miR-192-5p	Right rostral anterior cingulate	0.813	2.74E−06	0.038	17	−0.123	0.751	0.993	10	0.469	0.004	0.356
mir-664	Systolic blood pressure	0.995	3.18E−08	0.003	8	−0.179	0.838	1	4	0.934	4.52E−07	0.044

Pearson correlation analysis of miRNA expression levels in AUD subjects reveal significant relationships between specific miRNA expression levels and other medical, drinking and neuroimaging variables that were significantly different in AUD subjects

*R* Pearson correlation coefficient, *P-value* uncorrected P value for correlation, *BH FDR* Benjamini-Hochberg False Discovery Rate corrected P value, *N* number of pairs used in the calculation

#### miRNAs are associated with inferior frontal lobe, neuropsychological performance, drinking parameters and blood pressure

Many significant correlations found with miRNAs in Tiers A, B and C were observed with distinct regions of the brain, including those that have already been identified as primarily decreased in alcoholics, such as the left inferior frontal triangular gyrus (*mir*-*24*-*1*, *mir*-*136*, *mir*-*1976*) and a similar region in the right inferior frontal lobe along the pars opercularis (*mir*-*24*-*1*, *mir*-*136*). Notably, these two regions of the inferior frontal lobe are both involved in language function, and communicate with each other via the anterior corpus callosum, which we observed to be decreased in these same AUD subjects (Table 2). None of these particular miRNAs showed significant correlations with Letter Fluency Score. However, one miRNA (*miR*-*301a*-*3p*) did exhibit significant correlations with Letter Fluency Score and a region of the anterior inferior temporal lobe (the right anterior collateral transverse sulcus), although the functions of this cortical area are unclear at this time. The exploratory correlation analysis also identified some notable associations between miRNA levels and some of the drinking parameters (*let*-*7a*-*5p*, *mir*-*93*, *miR*-*139*-*5p*, *miR*-*1180*-*3p*), blood pressure (*mir*-*664*), and brain areas involved in higher order visual, sensory, and limbic processing (*mir*-*584*-*5p, mir*-*378a*, *mir*-*192*-*5p*, *mir*-*4433*; Table 5).

**Fig. 3:**
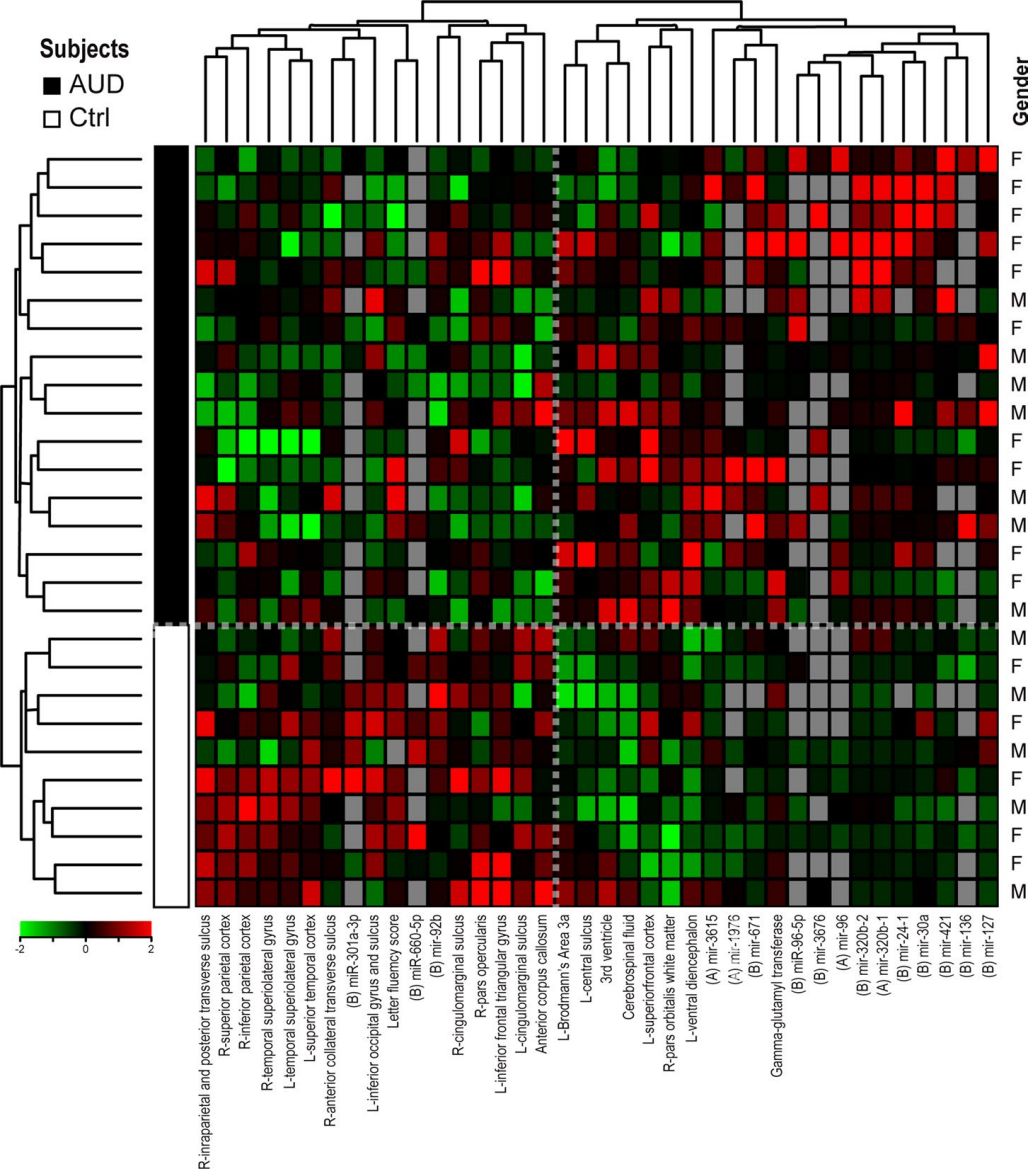
Variables and miRNAs delineate AUDs from controls. Hierarchical clustering of nominally significant clinical, neuropsychological and neuroimaging data combined with miRNAs belonging to Tiers A and B. Clusters reveal relationships between specific serum miRNAs and brain volumes. Log normalized median scaled values are shown. *L* left, *R* right

In addition to the correlational analyses of individual miRNAs and variables, we also used MEV4 software [38] to perform a hierarchical cluster analysis and identify patterns of correlations between sets of variables across subjects (Fig. 3). The Pearson clustering algorithm with average linkage distances was employed for this because it most effectively distinguished AUD and control subjects (Fig. 3, horizontal dashed line).

The clusters derived from this analysis, composed of mixed variable and miRNA components, provide additional insight into possible roles miRNAs play in the phenotypic changes we observe in AUD. For example, one cluster whose associations were not evident in the initial Pearson correlation analysis contained Tier A molecule *mir*-*92b* and several brain regions (left and right cingulomarginal sulci, left and right inferior frontal lobe areas, and the anterior corpus callosum). Notably, this cluster was located adjacent to another cluster containing Letter Fluency Score, as well as miR-660-5p (Fig. 3). *mir*-*92b has* been shown to be abundant in the developing cortex and to regulate the development of intermediate cortical progenitor cells [39]. While our AUD subjects were adults and thus not undergoing large scale cortical neurogenesis, these data nevertheless support a possible role for *mir*-*92b* in mediating brain damage resulting from AUD that will need further exploration because of the functional implications of the aforementioned brain areas regarding speech and language processing.

### Gene expression signatures related to brain-specific expression profiles

**Fig. 4:**
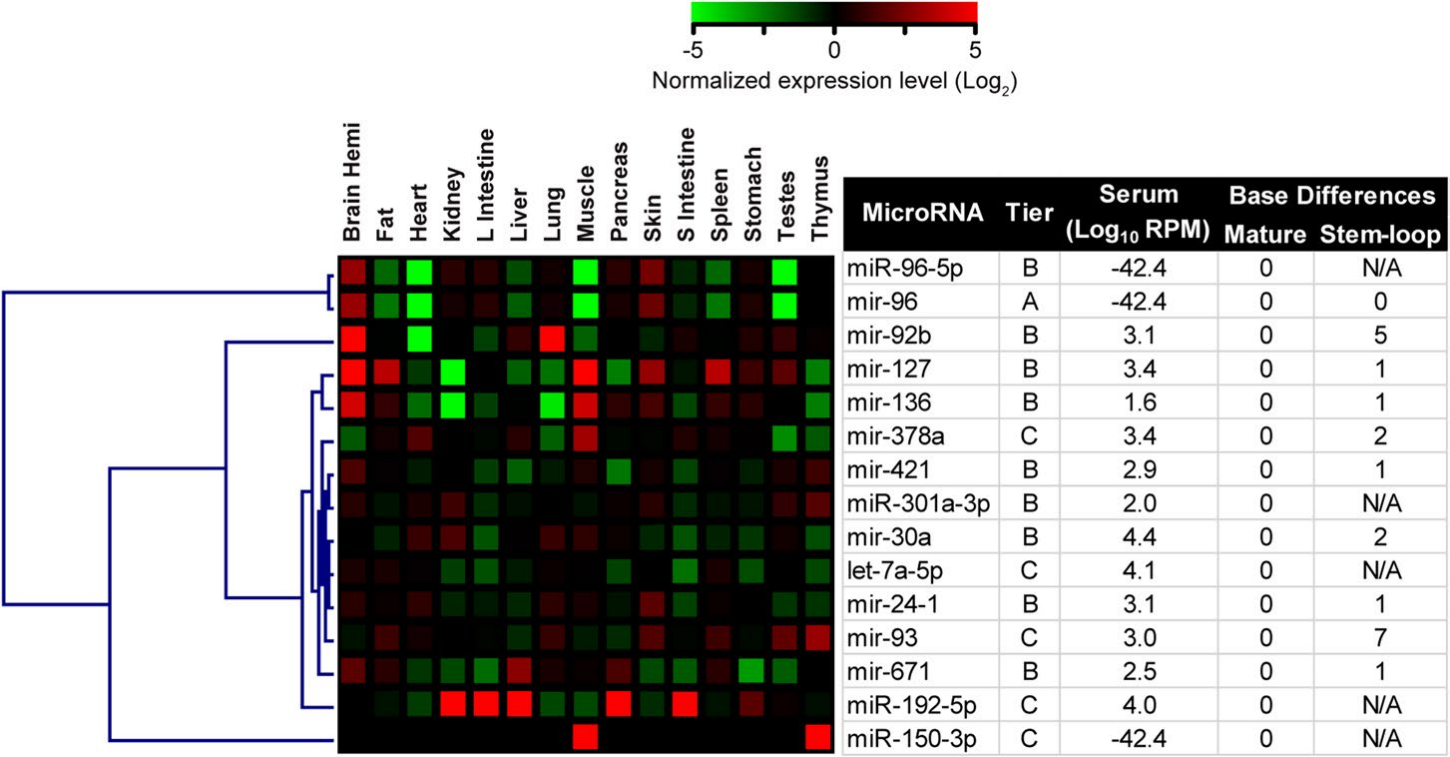
Comparison with tissue miRNA profiles suggests origins for specific serum miRNAs. Pooled miRNAs purified from tissues of 3 male P35 normal rats were compared to expression levels measured in whole brain hemispheres. *Rattus norgvegicus* expression levels for specific miRNAs belonging to Tiers A, B and C were clustered to determine microRNAs which are enriched in the brain compared to 14 other tissues. For relative comparisons, the levels of these miRNAs in the serum are also shown, and the number of base differences between the rat and human miRNA sequences are shown for both the fully mature and stem-loop forms

Although a peripheral biomarker can be useful from a purely diagnostic or correlative perspective, we also sought to obtain additional insight into the potential relevance of our serum-based miRNA data for providing information about brain miRNA levels. To begin to address this, we determined possible tissue origins for miRNAs of interest by performing comprehensive miRNA profiling on 15 distinct tissues and serum derived from three normal adolescent (P35) male rats. Tissue-specific profiles were generated on miRNAs from Tiers A, B and C. These data were normalized to the total miRNA levels in each tissue and compared to brain hemisphere levels in these same animals by hierarchical cluster analysis to discern meaningful patterns across tissues (Fig. 4). The levels of expression in the serum were also displayed for the same miRNAs.

Using this method, we found that most of the miRNAs of interest were present in the whole brain at moderate to high levels (except for *mir*-*378a*). Moreover, at least 5 of the miRNAs were grouped together following this analysis and appeared to be brain-enriched (*mir*-*96*-*5p*, *mir*-*96*, *mir*-*92b*, *mir*-*127*, *mir*-*136*; Fig. 4, upper). Notably, however, several of these were also enriched in muscle, spleen, skin, stomach, or testes, though in different combinations. Interestingly, *mir*-*92b* showed particular enrichment in brain and lung, which was not a common pattern among any other miRNAs. Most of the miRNAs in Fig. 4 were also highly expressed in the serum of normal adolescent rats, with the exception of mir-96, miR-96-5p, and miR-150-3p. Also of note was the fact that all of the miRNAs were completely conserved in their mature forms between rat and humans, although some nucleotide differences were present in the immature stem-loop sequences (Fig. 4, right). Thus, based on the cross-tissue analysis in rat, we are confident that at least some of the miRNAs in the human serum could be derived from the brain, although we cannot rule out other contributing sources without additional studies.

### Systems level cross-paradigm analysis reveals a common theme: miRNAs and targets that regulate cellular development, cell growth and proliferation, and cell death and survival are the most consistently affected in AUD subjects and drinking models

In addition to the human serum miRNA profiling, we also utilized two other ethanol exposure paradigms in an attempt to corroborate ethanol-induced effects found in human serum. We first performed serum miRNA profiling using an in vivo rat drinking model wherein adult rats were subjected to a 6.7 % ethanol (w/v) diet for 3 weeks. Exposures were either performed daily or 3 days per week. Serum miRNA levels were measured and compared to those from pair-fed controls who received an isocaloric non-alcoholic liquid diet during the same binge periods. To these data we then added additional miRNA profiling data obtained from an in vitro NS5 neural stem cell ethanol exposure model (400 mg/dL in the presence of FGF or TGFβ1 for 48 h) [40]. Because miRNAs have the capacity to modulate multiple gene targets and show cell context-dependent effects, we elected to perform a systems-level analysis rather than a screen for individual targets identified in our human studies. Thus, common molecular functions and biological networks were ascertained and compared across paradigms rather than individual miRNAs. The cross-paradigm systems level analysis was accomplished using QIAGEN Ingenuity^®^ IPA analysis using all nominally significant miRNAs (p < 0.1; see Additional file 1) from both the in vitro and in vivo ethanol exposure paradigms and human serum miRNAs from Tiers A and B (Table 6). Surprisingly, we found that 3 of the top 5 molecular functions represented in the altered miRNAs overlapped across these very different data sets: (1) Cellular development, (2) Cell growth and proliferation, and (3) Cell death and survival. These results provide strong evidence that that alcohol induces global miRNA expression changes affecting these functions in the mature brain, and potentially in developing brain cells as well. All of these changes are detectable in serum.

**Table 6:** In vivo and in vitro exposure paradigms converge with human data on same cellular functions

Humans			Rat drinking models						Mouse neural stem cells					
Serum			3 week daily serum			3 week binge serum			FGF2 + EtOH-treated			TGFβ1 + EtOH-treated		
Functional group	p-value	# Mol	Functional group	p-value	# Mol	Functional group	p-value	# Mol	Functional group	p-value	# Mol	Functional group	p-value	# Mol
Cellular development^a^	1.77 × 10^−3^	3	Cellular development^a^	1.10 × 10^−10^	32	Cellular development^a^	8.32 × 10^−18^	35	Cellular development^a^	2.64 × 10^−12^	49	Cell death and survival^a^	4.37 × 10^−7^	12
Cell death and survival^a^	2.36 × 10^−3^	4	Cell growth and proliferation^a^	1.10 × 10^−10^	27	Cell growth and proliferation^a^	8.32 × 10^−18^	34	Cell growth and proliferation^a^	9.86 × 10^−5^	42	Cellular development^a^	1.33 × 10^−5^	13
Cell morphology	4.72 × 10^−3^	1	Cell death and survival^a^	5.65 × 10^−8^	26	Cell death and survival^a^	6.89 × 10^−10^	27	Cell cycle	1.82 × 10^−4^	18	Cell growth and proliferation^a^	1.33 × 10^−5^	13
Cell function and maintenance	4.71 × 10^−3^	1	Cellular movement	1.15 × 10^−5^	18	Cell cycle	1.68 × 10^−7^	16	Cellular movement	1.82 × 10^−4^	27	Cell cycle	1.88 × 10^−5^	9
Cellular growth and proliferation^a^	1.47 × 10^−2^	2	Cell cycle	1.14 × 10^−4^	12	Cellular assembly and organization	5.94 × 10^−5^	5	Cell death and survival^a^	1.07 × 10^−3^	17	Cell morphology	1.85 × 10^−4^	5

Ingenuity IPA analysis of significantly changed miRNAs found using RNA-sequencing (Tiers A and B) show top functional groups affected by ethanol in each experiment. Different ethanol exposure paradigms, both in vitro (in neural stem cells) and in vivo (in humans and rats), show complete convergence for 3 of the top 5 functional groups (^a^) identified by IPA as affected by ethanol

**Fig. 5:**
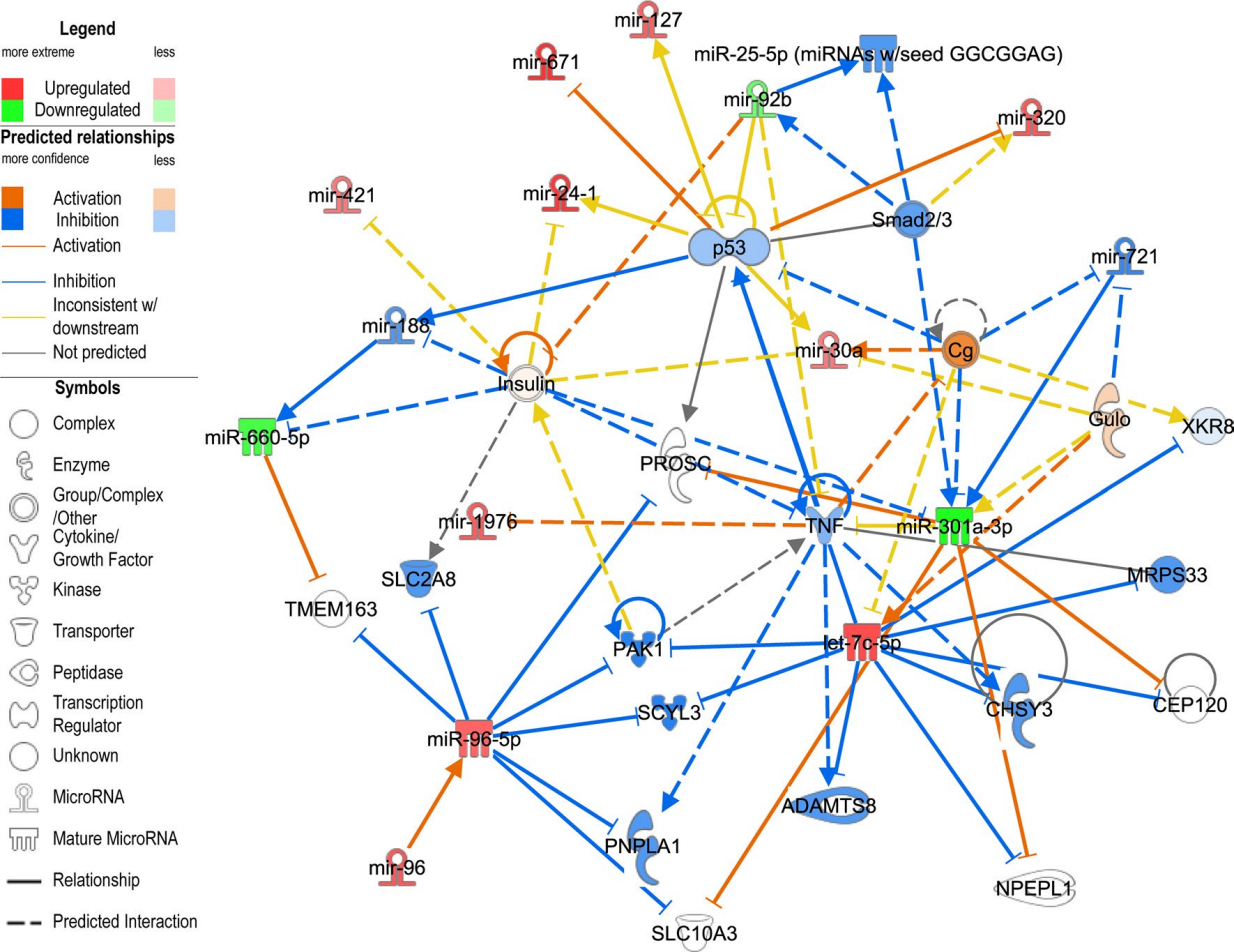
p53 is highly involved in AUD-related miRNA changes. Ingenuity IPA analysis of ethanol up- (*red*) or down- (*green*) regulated miRNAs in Tiers A & B show enrichment of molecules implicated in p53-related pathways. Molecular activity prediction shows a general inhibition of this pathway as a result of miRNA expression changes.* ADAMTS8* ADAM metallopeptidase with thrombospondin 8,* CEP120* centrosomal protein 120,* Cg* Choriogonadotropin,* CHSY3* chondroitin sulfate synthase 3,* Gulo* gulonolactone L-oxidase,* MRPS33* mitochondrial ribosomal protein S33,* NPEPL1* aminopeptidase-like 1,* PAK1* p-21 protein-activated kinase 1,* PNPLA3* patatin-like phospholipase domain-containing protein 3,* PROSC* proline synthetase,* SCYL3* SCY1-like 3,* SLC2A8* and 10A3, solute carriers 2A8 and 10A3,* TMEM163* transmembrane protein 163,* TNF* tumor necrosis factor,* XKR8* XK, Kell blood group complex subunit-related family 8

We further extended our systems-level analysis beyond the miRNAs themselves, to explore possible functional predictions in experimentally-confirmed or highly-predicted genes that are targeted by the miRNA molecules identified from human serum (Fig. 5). Inspection of the networks and nodes formed by the secondary (miRNA-gene) and tertiary (gene–gene) relationships revealed a few key hub molecules at the intersection of the common gene targets and networks affected by the miRNAs. The most striking of these hub molecules were p53 and tumor necrosis factor (TNF), which are critical mediators of cell cycle progression, DNA repair and apoptosis. Thus, these molecules may exert key influences over the miRNA-induced changes resulting from alcohol exposure.

## Conclusion

In this study, we examined the potential for serum miRNAs to serve as biomarkers of alcohol-related structural and functional CNS damage in human subjects. This diverse and largely uncharacterized class of genomic regulators has the capacity to enact change on a global scale. Harnessing several lines of inquiry, our work integrated medical, neuropsychological, neuroimaging and alcohol consumption measures with miRNA expression levels to probe for informative markers of ARBD.

Because we focused on younger individuals with AUDs, our work also adds to a small, but growing body of literature surrounding ARBD in young adults. Although we found significant group differences in our AUD subjects’ Letter Fluency test scores, we note that overall, AUD subjects did not show signs of cognitive impairment. This supports the notion that administering such tests on younger individuals may not prove to be as informative as they are for older chronic alcoholics with more pronounced deficits.

On the other hand, we observed more robust differences between AUD subjects and controls when we examined specific brain volumes. Furthermore, although many of our observations were similar to those reported previously in older alcoholic subjects, we observed these effects on younger subjects, with a significantly shorter history of heavy drinking. The importance of assessing alcohol-related brain damage in younger AUD subjects, even in the absence of obvious functional deficits, is only recently being explored [Reviewed in 41] and clearly merits further investigation. Regardless, however, such an approach is not feasible on a broad scale for monitoring due to its prohibitive costs.

In contrast, our results have identified potential miRNA biomarkers in the serum which ultimately could serve as practical, sensitive and reliable indicators of neurocognitive decline and ARBD. At this point at least, in the case of Tiers A and B microRNAs, we also found that the miRNA changes were observable using two complementary high-throughput quantification technologies. To be ideal, however, it would be helpful to know the time course over which such biomarker profiles develop, and whether such profiles were reversible or not as a result of abstinence from alcohol.

There are some limitations worth noting in the present study. First, the systems-level approach that we used to evaluate cellular functions takes into account the inherently pleiotropic nature of miRNAs. These miRNAs have the capacity to regulate hundreds of genes within any given cell. Thus, determining precise mechanisms of action clearly requires additional investigation. Further, interpretation of miRNA-mediated changes in gene expression also requires a combinatorial approach wherein they are analyzed in the context of accessible messenger RNA targets and the presence of other miRNA molecules that can synergize more drastic changes in gene expression.

In spite of these limitations, we have shown that alcohol acts on similar cellular functions: cell death and survival, cellular development and cell growth and proliferation across different ethanol exposure paradigms. Our analysis has revealed perturbation of biologically plausible pathways that have previously been shown to be altered in response to ethanol exposure in both prenatal and postnatal settings. Thus, although our results are by no means definitive, they highlight the tremendous potential of miRNAs as non-invasive ARBD biomarkers.

## Methods

### Subject selection and exclusion

All research performed on human subjects was approved by the Institutional Review Boards (IRBs) of SUNY Upstate Medical University and Crouse Hospital. Subjects were recruited from the Syracuse, New York area. Exclusion criteria included: age less than 18 or greater than 60, weight greater than 270 lbs, pregnancy, a history of head injury with loss of consciousness, co-morbid drug abuse (except for cigarette smoking) or co-morbid medical conditions including diabetes, cancer, hepatitis C, neurological diseases (such as seizure disorder) and major mental illness (except for anxiety and depression). All subjects had to agree to an MRI scan so patients with claustrophobia or metal implants of any kind were also excluded.

The Structured Clinical Interview for DSM-IV (SCID) [42] and Semi-Structured Assessment for the Genetics of Alcoholism version IV (SSAGA-IV) [43] were administered by a psychiatrist or psychiatric nurse practitioner to subjects to establish a diagnosis of DSM-IV alcohol dependence (AD) or alcohol abuse (AA), using widely-accepted criteria. The interview also collected demographics, past medical history as well as alcohol and tobacco use data. A total of 30 subjects participated in this study. Controls were selected from individuals who had not consumed a standard drink in the past month and who had never consumed more than two drinks at any time in their life. The final group of subjects were composed of 10 non-drinking controls and 20 currently diagnosed with AUDs, 16 with AD and 4 with AA.

### Medical, neuropsychological and neuroimaging assessments

Medical, neuropsychological and neuroimaging assessments were performed as described in Hicks et al. [3]. Briefly, after obtaining informed consent, subjects were screened using a dipstick urine test for drug abuse and a breath alcohol test. Subjects then underwent a brief neurological examination which screened for obvious signs of cerebellar damage (a common occurrence in chronic AUD subjects). Standardized neuropsychological tests were administered by trained examiners to evaluate cognitive function and included the Wechsler Abbreviated Scale of Intelligence (WASI), the Wechsler Memory Scale (WMS) and selected scales from the Delis-Kaplan Executive Function System [14], including trail-making tasks and word generation tasks such as letter fluency and category fluency tests.

Neuroimaging analysis was performed following a structural head MRI series obtained using a 1.5 T Philips Gyroscan scanner. Subjects were scanned in the sagittal plane using the following T-1 weighted inversion recovery 3D pulse sequence: TE = 4.6 ms; TR = 20 ms; 2 repetitions; matrix size 256 × 154 pixels; FOV 24 cm^2^; multishot = 32 repeats; TFE shortest possible pre-inversion time = 394 ms and 1.5 mm slice thickness.

We initially used the MRI data to screen our AUD subjects for evidence of neurodegenerative changes manifested as sulcal widening (a consequence of gray matter loss). To complete this process, skull-stripping and 3-D brain reconstruction were completed using MRIcro [44] and the built-in brain extraction tool [45]. The reconstructed volumes were then imported into Fiji [46] and subjected to both moments [18] and triangle [19] autothresholding algorithms to quantify total sulcal area in the dorsal brain surface. These sulcal areas were expressed as a percentage of the total dorsal surface area.

Based on the initial positive findings from our dorsal surface analysis, we completed a more comprehensive cortical reconstruction and volumetric segmentation of the MRI images using the semi-automated FreeSurfer image analysis suite (http://surfer.nmr.mgh.harvard.edu) [Reviewed in 20]. Measurements were made of parcellated volumes of more than 360 cortical and subcortical brain structures, including cortical gray matter, white matter, gyri and sulci. To control for overall brain shrinkage generally observed in brains of subjects with AUDs, volumes were normalized relative to the whole brain volume. All volumetric comparisons made in this study were between normalized whole brain ratios of specific regions.

For the serum miRNA studies, blood samples were collected into 10 cc BD Vacutainer^®^ SST™ Serum Separation Tubes, inverted several times, allowed to coagulate, then centrifuged according to manufacturer’s instructions. Serum samples were then frozen at −80 °C until needed for miRNA purification. To obtain the miRNA, the serum was centrifuged at 3000 rpm for 5 min and the cleared cell-free supernatant processed using the miRNeasy kit (Qiagen). An additional blood sample from each subject was also collected for routine laboratory measures that included (1) serum AST, ALT and GGT levels, (2) blood glucose levels, (3) differential white blood cell counts and (4) a complete blood cell count.

The combined medical, demographic, neuropsychological and neuroimaging data were first evaluated for equality of variances using a Fisher’s F ratio test. Notably, this use of the Fisher F test is a different use than what is determined in an analysis of variance F test for multiple groups. In this report, group differences for variables with unequal variances were determined using a Welch’s T-test. Otherwise, group differences were evaluated using a Student’s T-test.

### Rat drinking models

Animals were cared for in accordance with protocols approved by the Committee for the Humane Use of Animals (CHUA) at SUNY Upstate Medical University. A total of 38 (20 male and 18 female) Long-Evans rats obtained from Harlan Labs were used in the drinking studies. These rats were housed in individual cages and exposed to a 12 h reverse light/dark cycle. Fresh food was provided to all rats at 10:00 am. The liquid diet was obtained from OpenSource Research Diets™. Throughout the study duration, regular records of the rats’ body weights and ethanol (or control diet) consumption were maintained.

At postnatal day 29 rats were split into four groups (two treatment and two control groups). The first treatment and control groups examined the effects of daily drinking. These groups consisted of 11 rats each (6 male, 5 female). The treated rats were weaned onto an *ad lib* liquid ethanol-containing diet for 3 weeks, beginning in early adolescence and extending to early adulthood. Rats initiated the ethanol (ET) diet at a dose of 2.2 % v/v and were weaned up to 4.5 % v/v and finally 6.7 % v/v over a 3 day period. After this initial exposure, the rats received 6.7 % v/v liquid ethanol diet for three consecutive weeks. These treated rats were matched with pair-fed (PF) controls based on gender and initial body weight and received aliquots of the control diet defined by the amount of food consumed by the corresponding paired ethanol-fed rat. Maltose replaced ethanol in the control pair-fed diet to match for caloric and nutritional content. The next treatment and control groups examined the effects of intermittent binge drinking. These groups consisted of 8 rats each (4 male, 4 female). The treated rats received 6.7 % v/v liquid ethanol diet for three consecutive days each week, followed by 4 days of *ad lib* solid rat chow pellets (Purina). The PF controls for this group received the isocaloric maltose diet for 3 days, followed by 4 days of solid chow. After the 3 week ethanol exposure period, all rats in each group were euthanized with CO_2_ and blood was collected into BD Vacutainer^®^ SST™ tubes for serum miRNA isolation and routine clinical laboratory profiling, as described for the human sera. Analysis of blood tail vein samples from a separate cohort of rats treated with the same diet for 3 weeks indicated peak blood ethanol concentrations exceeding 300 mg/dL in both male and female adolescent rats that occurred within 3 h of access to the alcohol-containing diet at the beginning of each dark phase.

### Tissue miRNA profiling

To help determine the potential tissue origins of any candidate serum-based miRNA biomarkers, we performed a detailed analysis of miRNA levels in 15 different tissues and serum in a separate cohort of 3 untreated male Long Evans rats. These tissues included an entire brain hemisphere, hindlimb muscle, lung, heart, kidney, thymus, pancreas, subcutaneous abdominal fat, stomach, testes, liver, skin, spleen, large intestine and small intestine. Tissues were dissected and stored in RNAlater (Sigma), and subsequently homogenized prior to miRNA purification. Purified miRNAs from all three animals were pooled together to generate a specific miRNA profile for each tissue of origin, except for the brain hemispheres which were run individually.

### Mouse neural stem cells

Ethanol exposures of mouse NSCs were performed as described in Hicks et al. [4]. Briefly, eighteen 10 cm dishes coated with poly-l-ornithine hydrobromide and laminin (Sigma) were plated with 3.0 × 10^6^ cells and incubated for 24 h in a Euromed-N maintenance medium (Euroclone). Plates were then separated into 4 groups and exposed to different media for 48 h: maintenance media supplemented with 10 mg/ml FGF2 (Preprotech) (n = 4) or TGFβ1 (R&D Systems) (n = 5). In another set of dishes, 400 mg/dl of ethanol was added in each type of supplemented media (FGF2 + ethanol, n = 4 or TGFβ1 + ethanol, n = 5). All dishes were placed inside airtight containers with either sterile water alone or with 400 mg/dl ethanol to maintain constant concentration throughout the exposure period [42]. After the exposure, cells were harvested for miRNA purification.

### Nucleic acid preparation and expression profiling

Serum miRNAs from both humans and rats were purified using the miRNeasy Serum/Plasma kit (Qiagen). MicroRNAs from NSCs and all rat tissues were extracted using the miRNeasy Mini kit (Qiagen). The yield, purity and size distribution of the miRNA samples were assessed using a Bioanalyzer Nano RNA Lab Chip (Agilent).

NSC and rat serum miRNA samples were hybridized to GeneChip miRNA 2.0 arrays (Affymetrix). Human serum miRNA samples were hybridized to GeneChip miRNA 3.0 arrays (Affymetrix). RMA-normalized microarray data were analyzed using Partek Genomics Suite. For next-generation sequencing (NGS), human serum miRNAs and Rat tissue miRNA samples were used to prepare small RNA libraries using the TruSeq Small RNA Sample Prep kit (Illumina). Libraries were sequenced using a MiSeq Benchtop Sequencer (Illumina) and data uploaded into BaseSpace for initial QC and mapping.

FASTQ files were subsequently imported into Partek Flow and Strand NGS suites for analysis. Base calls below a phred score of 20 were trimmed. Reads were aligned to the human hg19 or rat Rn5 reference genomes using the Bowtie algorithm [47]. These were then quantified against both miRBase and RefSeq transcript miRNA annotations. Any miRNAs that were detected in fewer than five human subjects were discarded from further analysis. Raw miRNA counts were then normalized to the total number of miRNA reads per sample. Concentrations were expressed as a percentage of total miRNA reads. Notably, the described purification methods allowed sequencing of very pure miRNA samples, with more than 90 % of all reads in the sample attributed to miRNAs. All of the raw and processed microarray and RNA-sequencing data generated in this study have been deposited in the National Center for Biotechnology Information (NCBI) public Gene Expression Ominibus (GEO) database as SuperSeries GSE71579 and are available for immediate download.

The appropriate test for group differences between AUD and control expression levels was determined using the Fisher’s F test to determine unequal variances. For miRNAs with equal variances between groups, the Student’s T-test was used. If deemed unequal, the data for a specific miRNA was further tested for normality using the Shapiro–Wilk’s test. Group differences for miRNAs passing the normality test (p > 0.05) were determined using a Welch’s T-test while those that failed the normality test were compared using the Mann–Whitney test. Filtered human serum miRNAs were then designated into two different tiers depending on whether group differences and directional changes in expression levels were concordant with miRNA array data. Notably, these differences were not subjected to multiple testing correction because one of our main goals was to uncover quantitative relationships between variables that would clearly have been discarded had we used a stringent FDR correction for the between-group comparisons. The differences that are reported were designated as “nominally significant” for this reason. Lists of all differences in miRNA levels (p < 0.10) in the rat drinking paradigms and mouse neural stem cell cultures was also generated for comparison with the human data (Additional file 1). 

### Correlation, clustering and systems-level analyses

Pearson correlation matrices were generated for AUD, Control and combined subject groups. Comparisons were made between the combined medical, neuropsychological and neuroimaging variables and the normalized serum miRNA levels for each individual. For matrices composed of AUD subjects, we also included indices of drinking consumption for comparison with miRNA levels. The significance of these correlations was calculated using an R to T transformation and adjusted for multiple comparisons with the Benjamini-Hochberg False Discovery Rate (FDR) correction algorithm.

We combined all miRNAs from Tiers A and B with the nominally significant medical, neuroimaging and neuropsychological variables for hierarchical clustering. This was performed using the Microarray Experiment Viewer 4 (MEV4) software (TIGR, Johns Hopkins; http://www.tm4.org/mev.html) [38]. Data were scaled by mean-centering then division by the standard deviation of each variable. Clusters were created using the average linkage and the Pearson distance metric. The cross-tissue miRNA data from the rats were aligned and quantified as previously described. RPM normalized expression levels were then imported into MEV software, subjected to per-miRNA median centering and log_2_ transformation and used for clustering analysis to compare the expression levels across the 15 tissues for microRNAs in Tiers A, B (Table 5).

The miRNAs in Tiers A and B were analyzed using the Core Analysis workflow of QIAGEN Ingenuity^®^ IPA platform’s Core Analysis package to identify enriched gene networks considering only relationships that were highly predicted or experimentally observed. The top molecular and cellular functions identified in the AUD subjects, drinking rats, and in vitro studies of mouse NSCs were then compared.

## Additional file

Additional file 1: List of miRNAs showing changes (at p < 0.1 level) in rat drinking paradigms and mouse neural stem cell exposure paradigms.
